# Genus *Pempeliella* Caradja, 1916 and *P.bayassensis* Leraut, 2001 (Lepidoptera, Pyralidae) in Italy

**DOI:** 10.3897/zookeys.854.35351

**Published:** 2019-06-10

**Authors:** Manuela Pinzari, Mario Pinzari

**Affiliations:** 1 Dipartimento di Biologia, Università di Roma Tor Vergata, Via della Ricerca Scientifica 1, 00133, Roma, Italy Università di Roma Tor Vergata Rome Italy; 2 Piazza Francesco Morosini 12, 00136 Roma, Italy Unaffiliated Rome Italy

**Keywords:** distribution, *
matilella
*, *
ornatella
*, *
sororiella
*, sister species

## Abstract

*Pempeliellabayassensis* has been reported for the first time in Italy. This species has been confused with *P.ornatella* for a long time. Our study of the historical collections of Carlo Prola and Federico Hartig, and also newly collected materials from central Italy, allowed us to verify the presence of *P.bayassensis* in Italy. At present, this species is known only in central Italy (Lazio), where it coexists with *P.ornatella* and *P.sororiella*. We also provide information on the geographical distribution of the genus *Pempeliella* in Italy. In northern Italy we found *P.ornatella* and *P.sororiella*, and in the south (Puglia), *P.sororiella*. In Sardinia, *P.matilella*, which has been confused with *Delplanqueiacortella* in the past, coexists with *P.sororiella*.

## Abstract (it)

Viene segnalata per la prima volta in Italia *Pempeliellabayassensis*. Da sempre confusa con *ornatella*, per verificare la presenza in Italia della specie è stato effettuato lo studio delle collezioni storiche di Carlo Prola ed Federico Hartig e di materiale più recente raccolto in Italia centrale. Allo stato attuale, *P.bayassensis* sembra essere presente soltanto nel centro Italia ove convive con *ornatella* e *sororiella*. Inoltre diamo nota della distribuzione geografica in Italia delle specie appartenenti al genere *Pempeliella*. In particolare, nel nord sono presenti soltanto *P.ornatella* e *P.sororiella*. Nel sud è presente solo *sororiella* nelle Puglie. In Sardegna convivono *P.sororiella* e *P.matilella*, precedentemente confusa con *Delplanqueiacortella*.

## Introduction

In 2001 the genus *Pempeliella* Caradja, 1916 was adjusted (Leraut 2001a, 2001b) to include several European species: *P.ornatella* (Denis & Schiffermüller, 1775), which is present in almost all European countries and to Central Asia and Morocco; *P.lecerfella* (Lucas, 1933), in Morocco; *P.matilella* (Leraut, 2001), a Sardinian-Corsican endemism; *P.ardosiella* (Ragonot, 1887), in Spain, Gibraltar, and France but excluding Corsica; *P.sororiella* (Zeller, 1839), throughout southern Europe, from Spain to the Balkan Peninsula and also European Turkey; and *P.bayassensis*, in France and Morocco (Leraut 2001b), Spain (Gaston et al. 2014), and Switzerland (Schmid 2016). Leraut (2001a) excluded *Moitreliaitalogallicella* (Millière, 1883) and *Delplanqueiacortella* (Constant, 1884) from the genus *Pempeliella*. Slamka and Plant (2016) described as new *P.bulgarica* Slamka & Plant, 2016, from Bulgaria and also recorded it in Turkey and Hungary. It is externally closely similar to *Pempeliellasororiella* (Zeller, 1839), but easily distinguished by male and female genitalia.

In Italy, four species belonging to the genus *Pempeliella* were included in the checklist of the Italian fauna: *P.cortella*, *P.italogallicella*, *P.ornatella*, and *P.sororiella* (Bassi et al. 1995).

We have collected *P.bayassensis* since 1989 in an ongoing survey of the Lepidoptera fauna in central Italy (Pinzari et al. 2010; Pinzari 2009, 2016a, 2016b; Pinzari and Sbordoni 2013; Pinzari et al. 2013a, 2013b, 2015, 2016b, 2017b, 2018a, 2018b, 2018c, 2019). This species has been confused with *P.ornatella* for a long time, and *P.sororiella* is easily mistaken for *P.bulgarica* Slamka & Plant, 2016. According to Leraut (2001), to clarify our understanding of the distribution of *Pempeliella* species in Italy and also verify the accuracy of the historical faunistic information in the literature of the last century, we studied recently collected specimens from central Italy in ours and other private collections, and specimens from every Italian region preserved in the historical collections of the Museo civico di Zoologia di Roma and the Museo di Zoologia dell’Università la Sapienza di Roma.

We provide information on the geographical distribution of the genus *Pempeliella* in Italy and the first records of *P.bayassensis* in Italy.

## Materials and methods

### Species identification and distribution in Italy

We examined the collections of Mario Pinzari (Rome), Zerun Zerunian (Assisi), Carlo Prola (Museo civico di Zoologia di Roma, MCZR), and Federico Hartig (Museo di Zoologia dell’Università la Sapienza di Roma, MZUR).

For the taxonomic identifications of specimens, we examined either the external habitus (wingspan and wing pattern) or dissected the genitalia, using the characters reported by Leraut (2001, 2012, 2014), Leraut G.H.C. (2012), and Slamka and Plant (2016). Genital parts were glycerol-preserved in microtubes, which had their ends closed with vinyl glue; the microtubes were put under the specimens themselves.

To match up ancient specimens in the museum collections with the past literature, we recorded the collocation of the species (Hartig’s collection, boxes no. 17a and 18a; Prola’s collection, original collocation unknown) and the determination labels under the specimens. After our study, all specimens of the Hartig (MZUR) and Prola (MCZR) collections were returned to the museums and placed in a new collocation on the basis of our species determination. We added another determination label under the original label for each specimen.

To show an updated distribution of the genus *Pempeliella* in Italy, we mapped the collection sites of both the materials examined and records from the literature.

### History of the genus *Pempeliella* in Italy

Four species were included in the check list of Italian fauna (Bassi et al. 1995): *P.cortella* (northern Italy and Sardinia); *P.italogallicella* (northern Italy); *P.ornatellaornatella* (= *gigantella* Amsel, 1932; Italian Peninsula and Sicily); and *sororiellasororiella* (northern Italy, Sicily, and Sardinia).

Speidel et al. (2013) and Leraut (2014) provided data on *P.sororiella* in all Italian regions, including Sicily and Sardinia; *P.ornatella* in continental Italy and Sicily; and *P.matilella* in Sardinia only.

In the following text, we report the precise citations of the studied species mentioned in past papers.

***Pempeliellaornatella* (Denis & Schiffermüller, 1775)**

Valle d’Aosta: Parco Naturale Mont Avic: 1♂, 2 ♀♀, sentiero da Magazzino al Lago Selva, 1600–1800 m, 11.VII.1993; 4 ♀♀ sentiero da Covarey a Serva Desot, 1400–1600 m, 14.VII.1993, 22.VII.1994; 1 ♂, 1 ♀, dintorni di Covarey, 1200 m ca, 18.VII.1993, 23.VII.1994 (lux); 1 ♀, Serva Desot, prati, 1600 m ca, 24.VI.1995; (Baldizzone 1996).

Piemonte: Alpi Marittime, Viozene; Val Chisone, Fenestrelle, VII–VIII.1928; Alpi Biellesi, Piedicavallo, VII.1930; (Della Beffa 1941). S. Anna di Valdieri, dintorni del Lago sottano della Sella, 1900 m ca, 16.VII.1998; Terme di Valdieri, sentiero da Valasco superiore a Laghi Valscura e Claus, 2000–2300 m, 23.VII.1996, 30.VII.1997, 7.VIII.2001; Terme di Valdieri, Vallone del Gesso della Valletta, Pian della Casa, 1800 m, 24.VII.1997; Terme di Valdieri, Vallone del Gesso della Valletta, sentiero Pian della Casa al Colle del Mercantour, 1900–2200 m, 26.VII.2000; Entracque, Monte Ray, 1800 m, 20 and 24.VII.1999; Entracque, Valle della Rovina, Rocca Barbis, 1537–1800 m ca, 14.VII.1996, 20.VII.1997; S. Giacomo, vallone del Gesso della Barra: Gias Isterpis, 1380 m, 19.VII.1996 e sentiero per Rifugio Sori, 1600–1700 m ca, 19.VII.2000; Trinità, 1100 m, 24.VII.1996 (lux), 30.VII.1997 (lux), 13 and 14.VII.1998 (lux); Trinità, Vallone Grande, 1300 m ca, 15.VII.1996, 19.VII.1997, 13.VI.1999, 16.VI.2000; Trinità, sentiero per Colle della Garbella, 1550–1800 m, 30.VI.2000; Trinità, Valle del Sabbione, da Gias Ischietto a Gias dell’Adreit, 1200–1450 m ca, 13.VII.1996; (Baldizzone 2004). Parco Naturale delle Capanne del Marcarolo: Località, Cirimilla, Cascina Le Miniere, 300 m, 6.VI.2005 (lux); Strada-Cirimilla Capanne inferiori, 350 m, 26.V.2006 (lux); Cascina Cappellana, 450 m, 8.VI.2005 (lux), 21.VI.2005 (lux); Cascina Macerona, 500 m, 12.VI.2005 (lux), 21.VI.2005 (lux); Capanne inferiori, località Gli Olmi, 758 m, 9.VI.2003 (lux), 10.VI.2003 (lux), 19.VI.2003 (lux); (Baldizzone et al. 2013). Valdieri, luglio, agosto, (Turati and Verity 1911). Colle Fauniera (CN), Alpi Cozie, 3–6.VIII.2008, (Huemer 2009).

Veneto: Alpi Bellunesi, Mas, M. Piai, 450 m, 25.VII.1937; Alpi Agordine, Falcade, 6.VII.1932 (Rocca leg.); (Della Beffa 1941).

Trentino Alto Adige: Venezia Tridentina, Passo di Campolongo, 12.VII.1931 and S. Vigilio di Marebbe, 28.VI.1931 (Rocca leg.); Val S. Pellegrino, Someda, 15.VII.1938; (Della Beffa 1941). Brennero, Nord e Sud, (Hartig 1956). Tirolo, *Pempeliaornatella*, (Weiler (1877) in Burmann, 1995); Pempeliaornatellassp.gigantella, Venosta: Juval VI al L(ume) (Reitberger H. leg); Val d’Adige: S. Maurizio-Moritzing (Hellweger M. leg); Castel Firmiano-Sigmundskron. Val d’Isarco: Bressanone (Hellweger M. leg); Adamello: Malga Bedole (Biasoli in H.81); Val d’Amola 1800 m ♂♀ 29.VII.1924 (Tr); Fresine nell’VIII (Turati E. leg); Tonale ♂ 29.VI.1943 (Prola G., G. e Carlo leg); Ortler: Gomagoi ♂ 3.VII.1929 (Astfäller B. leg); Trafoi-Stelvio (Eppelsheim F., Wocke M., Frey leg); S. Valentino (Rocca); Tures-Taufers e Riva-Rain (W.80); Alpi Sarentine: Collalbo ♂ 12.VI, ♂♀ 17.VII, Rosswagen 1650 m ♂ 18.VI.1947 (Hartig); Avelengo-Hafling ♀ 23.VI, ♂ 17.VII.1930 (Hager K. leg); Dolomiti Ortisei-St. Ulrich 1.VII, Selva-Wolkenstein, Rif. Di Cisles-Regensburgerhutte 21.VII (Schawerda K. leg); Val di Non e Mendola: Tret ♂ 8.VI.1931, 17.VI.1932 (Castelli G. leg); Romeno ♀ 7.VI.1928 (Anonymous collector); Brenta e Paganella: Pinzolo 2 ♀♀ 30.VI and 11.VII.1926, Campiglio ♂ 1.VIII.1926, ♀ 21.VII.1927 (F); ibid. VII–VIII.1933–1935 (Hartig leg); Sette Comuni: Lavarone ♂ 19.VIII.1930 (Fiori A. leg); ♂ 17.VII.1933 (Anonymous collector); (Hartig 1958). Ritten (BZ), 1021 m, 1992–1995, 2000; Passo Lavazé (TN), 1790 m, 1992–1995); Huemer 2002. Schlern Nature Park, Castelrotto (BZ), (Huemer 2007).

Friuli Venezia Giulia: Alpi Carniche, Sappada, VII.1933; (Della Beffa 1941).

Emilia Romagna: Sestola, Appennino Emiliano (Turati 1923; In Parenti 1962). Croara, colline bolognesi (Parenti 1962). Toscana: *Pempeliaornatella* Schiff., Forte dei Marmi, (Verity 1904). Umbria: 1 ♂, Monte Subasio (PG), Fonte Bregno, 1000 m, 7.VI.2006; 1 ♂, Monte Subasio (PG), Colle S. Rufino, 1000 m, 13.V.2007; Z. & I. Zerunian leg. 2 ♂♂, Monte Subasio (PG), Mortaro Grande, 1200 m, 16.VI.2015; Z. Zerunian leg. (Pinzari et al. 2016).

Lazio: Borbona (RI) Fraz. Vallemare, 1 ♂, Colle Marcone, 1121 m, 16.VI.1989 (gen. praep. PIRA 274, M. Pinzari), 1 ♂, 1 ♀, idem, 18.VI.2007, 1 ♂, idem, 27.VI.2008, 1 ♂, 1 ♀, idem, 13.VI.2009, 1 ♂, idem, 26.VI.2009, 1 ♀, idem, 22.VII.2009, 1 ♂, idem, 18.VI.2010, 1 ♀, idem, 10.VII.2010; M. Pinzari leg; Posta (RI), Fraz. Villa Camponeschi, 1 ♂, Colle Petruccio, 1000 m, 19.V.2007, 1 ♂, idem, 20.V.2007; A. Zilli leg. Sightings. Colle Petruccio, 1000 m, 29.VI.2007, fide A. Zilli. (Pinzari et al. 2010). Abruzzo: La Maielletta, VII.1961 1 ♂; Passo Lanciano, VII.1960 1 ♂, 1 ♀, VII.1961 (6 ♂♂); (Parenti 1962).

***Pempeliellamatilella* (Leraut, 2001)**

Sardegna: Paratypes: 1 ♂, Italie, Sardaigne, Aritzo, “dint. Cant. Sa Casa”, 950 m, 24.VII.1936 (Conte Hartig) (prép. Gén. Leraut n° 6639; MNHN, Paris; 3 ♀♀, mêmes coordonnées, 1 ♀, Sardaigne, Aritzo, 3.VIII.1936 (H.G. Amsel); (Leraut 2001).

***Pempeliellasororiella* (Zeller, 1839)**

Piemonte: Terme di Valdieri, Vallone del Gesso della Valletta, sentiero Pian della Casa al Colle del Mercantour, 1900–2200 m, 26.VII.2000, (Baldizzone 2004).

Trentino Alto Adige: Trentino Alto Adige, Val Venosta, S. Valentino alla Muta, m. 1500 e Burgusio, m. 1300, VI.1939, (Della Beffa 1941). Brennero Sud, (Hartig 1956). Tirolo, *Pempeliellasororiella*, (Hellweger 1929 in Burmann 1995). Tirolo, Hartig *Pempeliellasororiella* (Burmann 1995); Dolomiti: 1 ♂ Schluderbach, 1 ♀, Val Popena (Mann In Mus. Vind. Htg) (Hartig 1958).

Friuli Venezia Giulia: Tagliamento, Cornino, 180 m, Peonis, Avasinis, 250 m. (Deutsch 2006).

Lazio: Fondi, S. Anastasia, 1 ♂, 1–12.VIII, Predota C. leg. (Hartig 1939). Borbona (RI) Fraz. Vallemare, 1 ♂, Colle Marcone, 1121 m, 2.VIII.2012, 1 ♂, idem, 15.VIII.2012 (gen. praep. PYRA 259, M.Pinzari), M. Pinzari leg. (Pinzari et al. 2013b).

Sicilia: Siracusa, giugno, Zeller leg. (Curò 1880; Minà Palumbo and Failla-Tebaldi 1889). Zappulla (ME), VII (Mariani, 1939).

Sardegna: Aritzo 29.VII, Sa Casa 24–29.VII. 1936, Strada per Desulo 8.VII. 1936, (Hartig and Amsel 1951).

***Pempeliellacortella* (Constant, 1884)**

Sardegna: Sa Casa 24–29.VII.1936; Aritzo 9–27.VII.1936; Piano di Sadali, 5.VII.1936; Strada per Desulo, 8.VII,.1936 (Hartig and Amsel 1951). This citation was attributed *P.matilella* after the study of Hartig’s collection by Pinzari and Pinzari (in press). At present, *P.cortella* (= *Delplanqueiacortella*) has not been revealed yet in Sardinia.

## Results

We identified the species of *Pempeliella* in Italy and grouped the specimens following the publication by Leraut (2001). Our study of Hartig’s and Prola’s collections revealed that the specimens collected in Trentino (*N* = 15) and Liguria (*N* = 2) were *P.ornatella* and that the specimen from Puglia was *P.sororiella*.

In central Italy we found that there were only three of the four Italian species of *Pempeliella*: *P.ornatella*, *P.bayassensis* and *P.sororiensis*. In Latium, we collected: five males and three females of *P.bayassensis*; 13 males and 14 females of *P.ornatella*; two males and one female of *P.sororiella*. From Sardinia, four specimens of *P.sororiella* and 27 of *P.matilella* were identified in Hartig’s collection.

### Materials examined and newly collected

***Pempeliellaornatella* (Denis & Schiffermüller, 1775)**

Liguria: 1 ♂, (gen. praep. PIRA 534, M. Pinzari), Alpi Marittime, Carmo Langan, 14.VIII.1950, Prola leg. MCZR; 1 ♀, (gen. praep. PIRA 535, M. Pinzari), Alpi Liguri, Colla Melosa, 17.7.1985, (legit absent), MCZR; 1 ♂, (abdomen absent), Alpi Liguri (IM), Colla Melosa, 16.VII.1983, V. Raineri leg, MCZR. Piemonte: 1 ♀, (abdomen absent), Alpi Cozie, Sestriere, 23.VII.1950, Prola. Lombardia: 1 ♂, (gen praep. PIRA 519, M. Pinzari), colloc. *gigantella*, Alpi del Tonale, 29.VI.1943 Prola MCZR (Fig. 3 B).

**Figure 1.**
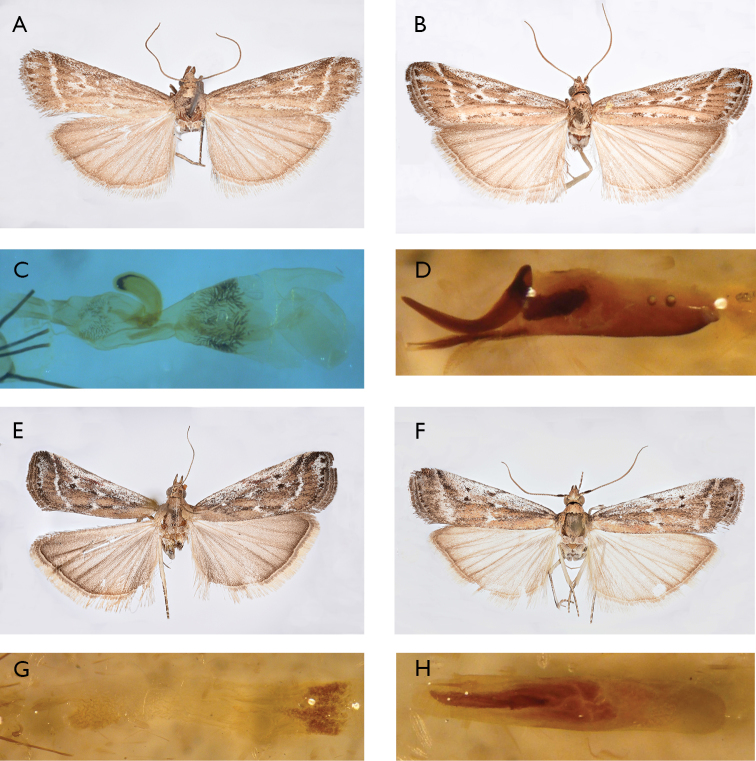
*Pempeliella* species in Italy. **A***P.ornatella* ♀ (wingspan 21 mm) **B***P.ornatella* ♂ (wingspan 24 mm) **C***P.ornatella* ♀ bursa copulatrix (gen. praep. PIRA 515) **D***P.ornatella* ♂ aedeagus (gen. praep. PIRA 517) **E***P.bayassensis* ♀ (wingspan 21 mm) **F***P.bayassensis* ♂ (wingspan 24 mm) **G***P.bayassensis* ♀ bursa copulatrix (gen. praep. PIRA 280) **H***P.bayassensis* aedeagus (gen. praep. PIRA 277).

Trentino Alto Adige: 2 ♂♂, (gen. Praep. PIRA 513, PIRA 516, M. Pinzari), Mad. di Campiglio, 13.VII.1933, and coll. Cte Hartig; 1 ♂, (gen. praep. PIRA 514, M. Pinzari), M. di Campiglio, Trentino, Italia, 1522 m, 20.VII.1933, 1 ♀, (gen praep. PIRA 515, M. Pinzari, Fig. 1A, C, idem, 10.VIII.1933, and coll. Cte. Hartig; 1 ♂, (gen. praep. PIRA 517, M. Pinzari, Fig. 1B, D), Alpi di Merano, Hafling, 900 (m), 2.VI.1931. All colloc. *gigantella*, Coll. Hartig MZUR. 1 ♂, M. di Campiglio, Trentino, Italia, 1522 m, 21.VII.1933, 1 ♂, idem, 15.VII.1933, and coll. Cte Hartig; 3 ♂♂, Mad. di Campiglio, 13.VII.1933, and coll. Cte Hartig; 1 ♂, determination label by E. Turati (Fig. 3B)., Mad. di Campiglio, 1515 m, 10.VII.1933, and coll. Cte Hartig (Fig. 3B); 1 ♂, M. di Campiglio Pfeiffertafel, 1600 (m), 13.VIII.1933, and coll. Cte Hartig. 1 ♀, Mad. di Campiglio, Via degli Orsi, 24.VII.1933, and coll. Cte Hartig; M. di Campiglio, 1522 m, 1 ♂, 27.VI.1935, 1 ♂, idem, 8.VII.1935, coll. Cte Hartig; 1 ♀, Mad. di Campiglio, 1515 m, 10.VII.1933, and coll. Cte Hartig, MZUR.

Lazio: 1 ♂, (gen. praep. PIRA 522, M. Pinzari), colloc. *gigantella*, M. Terminillo, m 1800, 16.VII.40 Prola leg. Coll. Hartig MZUR. Borbona (RI) Fraz. Vallemare, 1 ♂, (gen. praep. PIRA 274, M. Pinzari), Colle Marcone, 1121 m, 16.VI.1989, 1 ♂, 1 ♀, idem, 18.VI.2007, 2 ♂♂, 1 ♂, idem, 26.VI.2009, 1 ♀, idem, 22.VII.2009, 1 ♂, idem, 18.VI.2010, 1 ♀, idem, 10.VII.2010, 1 ♂, idem, 22.V.2011, 1 ♂, idem, 25.VI.2011, 1 ♀, idem, 8.VI.2012, 1 ♂, idem, 15.VI.2012, 1 ♂ (gen. praep. PIRA 278, M. Pinzari), idem, 16.VI.2012 1 ♀, idem, 22.VI.2012, 1 ♂, idem, 19.VI.2013, 1 ♀, idem, 5.VII.2013, 1 ♂, idem, 20.V.2014, 1 ♀, idem, 7.VI.2014, 1 ♀, idem, 7.VII.2014, 2 ♀♀, idem, 24.VI.2016, 1 ♂, 1 ♀, idem, 9.VII.2016, 1 ♀, idem, 19.VIII.2016, 1 ♀, idem, 17.VI.2017; M. Pinzari leg. Posta (RI) Fraz. Villa Camponeschi, 1 ♂, Colle Petruccio, 1000 m, 19.V.2007, 1 ♂, idem, 20.V.2007; A. Zilli leg.

Abruzzo: 1 ♂, (gen. praep. PIRA 521, M. Pinzari), colloc. *ornatella*, Abruzzo, Collelongo, m 1300, 28.VI.75, Prola leg. MCZR. 1 ♂, (gen. praep. PIRA 536, M. Pinzari), Abruzzo, Tufo, m. 900, 28.VI.75 Prola leg. MCZR.

***Pempeliellamatilella* Leraut, 2001**

**Figure 2.**
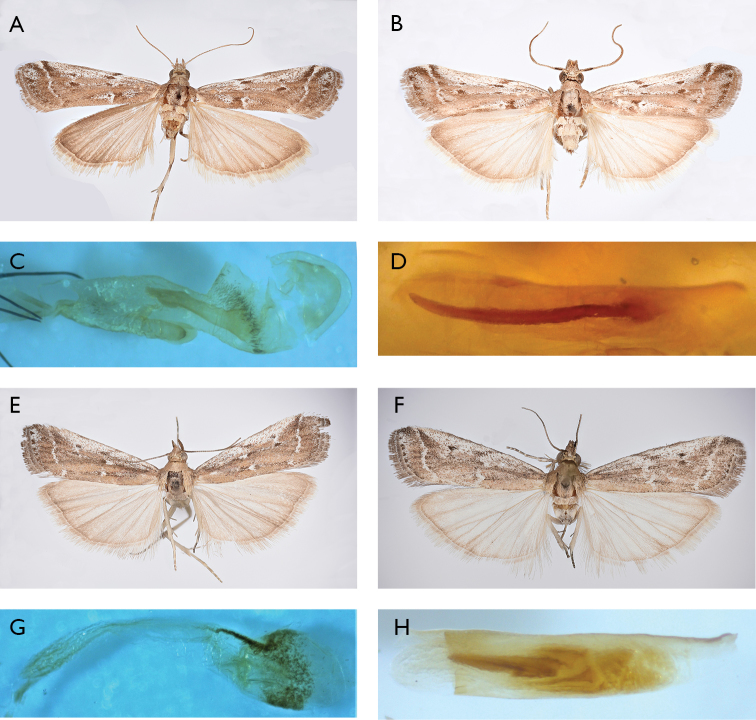
*Pempeliella* species in Italy. **A***P.matilella* ♀ (wingspan 22 mm) **B***P.matilella* ♂ (wingspan 22 mm) **C***P.matilella* bursa copulatrix (gen. praep. PIRA 488) **D***P.matilella* aedeagus (gen. praep. PIRA 490) **E***P.sororiella* ♀ (wingspan 14 mm) **F***P.sororiella* ♂ (wingspan 20 mm) **G***P.sororiella* bursa copulatrix (gen. praep. PIRA 282) **H***P.sororiella* aedeagus (gen. praep. PIRA 537).

**Figure 3.**
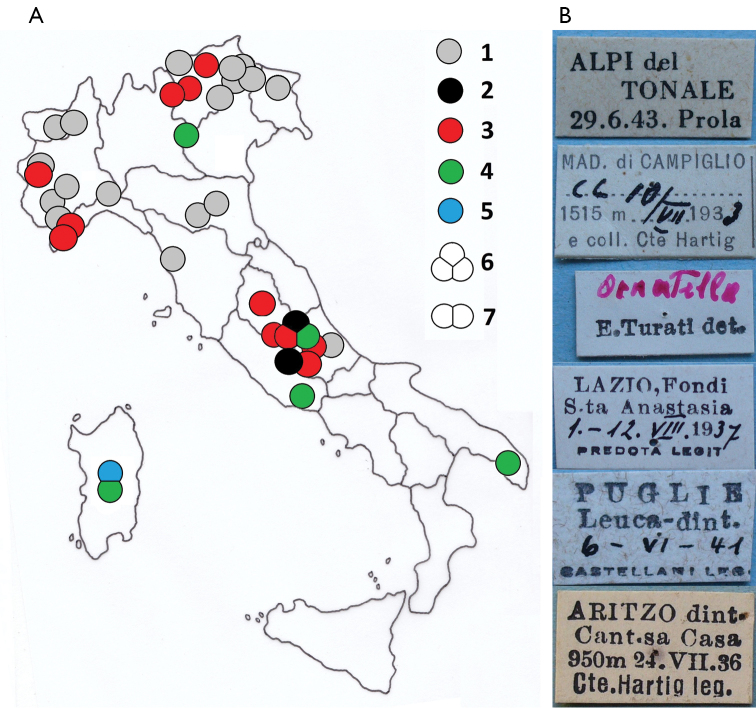
*Pempeliella* species in Italy. **A** Distribution of genus *Pempeliella* Caradja, 1916 in Italy: 1) unverified quotations; 2) *P.bayassensis*; 3) *P.ornatella*; 4) *P.sororiella*; 5) *P.matilella*; 6) three and two (7) coexisting species. **B** Some historical entomological cards of the specimens in the map by: 1) Prola; 2) Hartig; 3) Turati; 4) Predota; 5) Castellani; 6) Hartig, are shown to the right of the map.

Sardegna: 1 ♀, Sard. centr., Aritzo, 6.VII.1936, 1 ♂, (gen. praep. PIRA 493, M. Pinzari), idem, 11.VII.1936, 1 ♀, (gen. praep. PIRA 488, M. Pinzari, Fig. 2A, C), idem, 28.VII.1936; 3 ♂♂, (gen. praep. PIRA 490, M. Pinzari, Fig. 2B, D), 2 ♀♀, (gen. praep. PIRA 487, M. Pinzari), Aritzo dint., Cant.sa Casa, 950 m, 24.VII.1936 (Fig. 3B); 2 ♂♂, (gen. praep. PIRA 491, M. Pinzari), idem, 29.VII.1936; all Cte Hartig leg.

***Pempeliellabayassensis* Leraut, 2001**

Lazio: 1 ♂, (gen. praep. PIRA 520, M. Pinzari), Colloc. *subornatella*, Lazio, M.te Flavio, 800 m, 30.V.1938, Hartig legit. MZUR. Borbona (RI), Fraz. Vallemare: 1 ♀, (gen. praep. PIRA 275, M. Pinzari), Colle Marcone, 1121 m, 27.VI.2008, 1 ♂, (gen. praep. PIRA 281, M. Pinzari), idem, 9.VII.2011, 1 ♀, (gen. praep. PIRA 280, M. Pinzari, Fig. 1E, G), idem, 24.VIII.2011, 1 ♂, (gen. praep. PIRA 277, M. Pinzari, Fig. 1F, H), idem, 18.V.2012, 1 ♂, idem, 19.VI.2013, 1 ♂, (gen. praep. PIRA 541, M. Pinzari), idem, 2.VI.2014, 1 ♀, idem, 6.VI.2014, 1 ♀, idem, 24.VI.2016; 1 ♀, (gen. praep. PIRA 276, M. Pinzari), Bivio Brignola, 1061 m, 1.VI.2012, M. Pinzari leg.

***Pempeliellasororiella* (Zeller, 1839)**

Veneto: 1 ♂, (gen. praep. PIRA 499, M. Pinzari), Lago di Garda, Torri Benaco, 6.VI.1940, Hartig leg. MZUR.

Lazio: 1 ♂, (gen. praep. PIRA 555, M. Pinzari), Lazio, Fondi S.ta Anastasia, 1–12.VIII.1937, Predota leg. (Fig. 3B) MZUR. Borbona (RI) Fraz. Vallemare: 1 ♀, (gen. praep. PIRA 282, M. Pinzari), Colle Marcone, 1121 m, 2.VIII.2012, 1 ♂, (gen. praep. PYRA 259, M. Pinzari), idem, 15.VIII.2012, 1 ♂, (gen. praep. PYRA 537, M. Pinzari, Fig. 2F, H), idem, 29.VIII.2015, M. Pinzari leg.

Puglie: 1 ♀, (gen. praep. PIRA 554, M. Pinzari, Fig. 2E, G), Puglie, Leuca dint. 6.VI.1941 Castellani leg (Fig. 3B), MZUR.

Sardegna: 1 ♀, Sard. centr., Strada Desulo, 650 m, 8.VII.1936, Cte Hartig leg; 1 ♀, (gen. praep. PIRA 539, M. Pinzari), Aritzo, dint. Cant.sa Casa, 950 m, Cte Hartig leg. MZUR. 1 ♀, Sard. centr. Aritzo, 29.VII.1936, Cte. Hartig leg; 1 ♀, Aritzo, dint. Cant.sa Casa, 950 m, Cte Hartig leg. MZUR.

### Distinguishing species

***Pempeliella ornatella***

We examined 48 specimens of *P.ornatella* that were collected from northern and central Italy. In general, males and females of *P.ornatella* differed in wingspan (Mann-Whitney U test, wingspan, *N_males_* = 29, *N_females_* = 19, *U* = 30.00, *Z*_adj_ = 5.26, *p* < 0.00001). Wingspan values were on average equal to 24.48 mm ± SE 0.27 (*N* = 29, range: 21–27, SD = 1.45) in males and to 21.16 mm ± SE 0.31 (*N* = 19, range: 18–24 mm, SD = 1.34) in females. Sexual dimorphism in wingspan was present when considering specimens of northern and central Italy either separately or as a whole (northern specimens: Mann-Whitney U test, wingspan, *N_males_* = 13, *N_females_* = 4, *U* = 6.50, *Z*_adj_ = 2.24, *p* = 0.000007; central specimens: Mann-Whitney U test, wingspan, *N_males_* = 15, *N_females_* = 15, *U* = 6.00, *Z*_adj_ = 4.51, *p* = 0.025).

The specimens of *P.ornatellagigantella* collected in the northern (n) Italy showed values of wingspan higher than *ornatella* in central Italy (*cI*) (*males*: Mann-Whitney U test, wingspan, *N_n_* = 12, *N_cI_* = 15, *U* = 50.5, *Z*_adj_ = 1.99, *p* = 0.046; *females*: Mann-Whitney U test, wingspan, *N_n_* = 4 , *N_cI_* = 15, NS).

***Pempeliella bayassensis***

We examined nine specimens of *P.bayassensis*. This species could be easily confused with *P.ornatella* due to their very similar habitus, and the two species are sympatric and coexist in central Italy. The size of *P.bayassensis* is smaller than *P.ornatella*. The wingspan showed a greater mean value in *P.ornatella* than in *P.bayassensis* (mean value ± standard error: males, 23.33 ± 0.67 mm, SD = 1.15, *N* = 3, range 22–24 mm; females, 21.67 ± 0.49 mm, SD = 1.21, *N* = 6, range 20–23). *P.bayassessis* has a distinctive forewing post median line that is more curved in *bayassensis* than in *ornatella* (Fig. 1A, B, E, F). Accordingly, it could be identified only by external habitus. However, in doubtful cases, *P.bayassessis* can be easily recognized by characters of the genitalia (Fig. 1C, D, G, H).

***Pempeliella sororiella***

We examined 10 specimens of *P.sororiella*. This species can be easily confused with *P.bulgarica* due to their very similar habitus (Slamka and Plant 2016), but our examination of genitalia confirmed the species as *P.sororiella* (Fig. 2G, H).

### Distribution of species in Italy

The distribution map of materials examined shows that all moths from Trentino, Liguria, Emilia Romagna, and Toscana are *P.ornatella* (Fig. 3A). *Pempeliellasororiella* was found in northern Italy but never together with *P.ornatella*. In central Italy, we found three species, *P.ornatella*, *P.bayassensis*, and *P.sororiensis*. These species coexist in Latium, but *P.sororiella* and *P.bayassensis* were infrequent and difficult to sample by lamp. In eastern Latium, a single specimen of *P.bayassensis* (gen. praep. PIRA 520, M. Pinzari) was sampled in 1938 by Hartig at Monte Flavio. In Umbria and Abruzzi, all specimens were *P.ornatella*. In southern Italy (Apulia), there was only one specimen of *P.sororiella*, which was found in 1941 by Omero Castellani; it is preserved in Hartig’s collection (MZUR). Currently, no species of *Pempeliella* have been recorded in Sicily. Finally, *P.matilella*, which was erroneously confused with *Delplanqueiacortella*, and *P.sororiella* were found to be in Sardinia.

## Discussion

The historical collections studied include moths collected by several entomologists and in various regions of Italy. Our study of these collections and the published literature on *Pempeliella* allowed us to reconstruct the distribution of *Pempeliella* species in Italy. Our study revealed that *P.ornatella* is present in northern and central Italy, that *P.sororiella*, although is a less frequent species in Italy, is present through the peninsula and in Sardinia, that *P.matilella* is known only in Sardinia (Pinzari and Pinzari in press), and that *P.bulgarica* is not present in Italy. Finally, the main novelty of this paper is that *P.bayassensis*, which is present only in central Italy, is reported from Italy for the first time. Federico Hartig collected a single specimen of *P.bayassensis* in 1938 but it was misidentified as *P.ornatella*. We have since collected this species at Vallemare (Rieti) in Latium, where *P.bayassensis* coexists with *P.sororiella* and *P.ornatella*. At present, *P.bayassensis* has not been recorded yet in other localities in Italy.
